# Genetic Analysis and Serological Detection of Novel O-Antigen Gene Clusters of *Plesiomonas shigelloides*

**DOI:** 10.4014/jmb.2010.10008

**Published:** 2021-02-08

**Authors:** Xiaochen Wang, Daoyi Xi, Yuehua Li, Junxiang Yan, Jingyun Zhang, Xi Guo, Boyang Cao

**Affiliations:** 1 Key Laboratory of Molecular Microbiology and Technology of the Ministry of Education, TEDA College, Nankai University, Tianjin 300457, P.R. China; 2 TEDA Institute of Biological Sciences and Biotechnology, Nankai University, Tianjin 300457, P.R. China; 3 Tianjin Key Laboratory of Microbial Functional Genomics, TEDA College, Nankai University, Tianjin 300457, P.R. China; 4 State Key Laboratory for Infectious Disease Prevention and Control, National Institute for Communicable Disease Control and Prevention, Chinese Center for Disease Control and Prevention, 155 Changbai Road, Changping District, Beijing 102206, P.R. China

**Keywords:** *Plesiomonas shigelloides*, O-antigen gene clusters, serogroups, PCR assay

## Abstract

*Plesiomonas shigelloides*, a member of the family *Vibrionaceae*, is a gram-negative, rod-shaped, facultative anaerobic bacterium with flagella. *P. shigelloides* has been isolated from such sources as freshwater, surface water, and many wild and domestic animals. *P. shigelloides* contains 102 Oantigens and 51 H-antigens. The diversity of O-antigen gene clusters is relatively poorly understood. In addition to O1 and O17 reported by other laboratories, and the 12 O serogroups (O2, O10, O12, O23, O25, O26, O32, O33, O34, O66, O75, and O76) reported previously by us, in the present study, nine new *P. shigelloides* serogroups (O8, O17, O18, O37, O38, O39, O44, O45, and O61) were sequenced and annotated. The genes for the O-antigens of these nine groups are clustered together in the chromosome between *rep* and *aqpZ*. Only O38 possesses the *wzm* and *wzt* genes for the synthesis and translocation of O-antigens via the ATP-binding cassette (ABC) transporter pathway; the other eight use the Wzx/Wzy pathway. Phylogenetic analysis using *wzx* and *wzy* showed that both genes are diversified. Among the nine new *P. shigelloides* serogroups, eight use *wzx/wzy* genes as targets. In addition, we developed an O-antigen-specific PCR assay to detect these nine distinct serogroups with no cross reactions among them.

## Introduction

*Plesiomonas shigelloides*, a member of the family *Vibrionaceae*, is a rod-shaped, gram-negative facultative anaerobic bacterium with flagella. *P. shigelloides* has been isolated from such sources as freshwater, surface water, and many wild and domestic animals [1]. Infection with *P. shigelloides* has been associated with travel to or residence in tropical and subtropical countries and with the ingestion of raw seafood, particularly oysters. Despite the epidemiological association, the causal relationship between *P. shigelloides* and diarrhea remains to be confirmed [2].

Aldová *et al*. recognized 102 O-antigens and 51 H-antigens of *P. shigelloides* [3]. *P. shigelloides* strain 302-73, which was originally isolated in Japan [4], was determined and titled as the ‘O1 representative strain.’ In 2010, the complete chemical structures of its O-antigen and lipopolysaccharide (LPS) core were characterized [5]. The whole genome was sequenced in 2013 and deposited in GenBank (AQQO01000000) [6]. *P. shigelloides* O17, which carries the same O-antigen as *Shigella sonnei*, was used in vaccine production against shigellosis [7]. Our previous study reported the diversity of *P. shigelloides* O-antigen gene clusters, and also established an identification and detection method for molecular serotyping [8]. The variability of the O-antigen provides the primary basis for serotyping schemes for *P. shigelloides* [9]. Based on the 12 O-antigen clusters of *P. shigelloides*, serogroups O2, O10, O12, O23, O25, O26, O32, O33, O34, O66, O75, and O76, it became clear that the O-antigen gene clusters group together and are located on the chromosome between the *rep* and *aqpZ* genes. Among these 12 serogroups, nine use the Wzx/Wzy pathway to synthesize and translocate O chains, and the other three use the *wzm/wzt*-encoded ATP-binding cassette (ABC) transporter pathway [10]. Furthermore, a suspension array that detected 12 distinct O-antigens was developed [8]. In the present study, nine new O-antigen gene clusters of *P. shigelloides* (O8, O17, O18, O37, O38, O39, O44, O45, and O61) were sequenced and annotated. Like the 12 previously identified serogroups, the genes for the O-antigens group together in chromosomes between *rep* and *aqpZ*, which encode a helicase and an aquaporin, respectively. Only O38 possesses the *wzm* and *wzt* genes for the synthesis and translocation of O chains via the ABC transporter pathway, and the other eight use the Wzx/Wzy pathway.

The identity of Wzx/Wzy and Wzm/Wzt is low (30-50%); therefore, they are used as determinants for each serovar and have the potential to be DNA molecular markers for serotyping. In our previous work, *wzx* and *wzy* were used to serotype *Escherichia coli*, *Shigella* spp. [11], *Salmonella* spp. [12], and *Pseudomonas aeruginosa* [13]; *wzm* and *wzt* were used to serotype *Legionella pneumophila*; and *wzx*, *wzy*, and *wzm* were used to serotype *P. shigelloides* [8]. The phylogenetic analysis of *wzx* and *wzy* showed that both of these genes are diversified and are potential candidates for molecular serotyping. Thus, out of the nine *P. shigelloides* serogroups, eight employed *wzx*/*wzy*/*wzm* genes as targets.

In the present study we used the PCR assay as the detection technique. The PCR has several advantages in terms of safety, saving on cost/time, high specificity, sensitivity, and reproducibility. We show that all nine new *P. shigelloides* serogroups could be detected without any cross reactions among them.

## Materials and Methods

### Strains and Genomic DNA Extraction

The *P. shigelloides* reference strains used in this study are shown in Table 1. All the strains were cultured in Tryptone Soya Broth (TSB) (QingDao ShuiRi Bio-Technologies Co., Ltd., China) at 37°C with aeration at 200 rpm. Genomic DNA was extracted from 1.5 ml of the overnight bacterial cultures (approximately 10^8^ CFU/ml) using a DNA extraction kit according to the manufacturer's instructions (CWBiotech, China).

### Library Construction

A gene sequencing library was constructed as previously described [8]. Briefly, DNA was fragmented by nebulization, end-repaired, and phosphorylated. An A-tail and adaptors were ligated to the end-repaired DNA fragments according to the manufacturer’s instructions (Illumina Inc., United Kingdom).

### Genome Sequencing, Assembly, and Annotation

According to a previous report [14], the whole genomes of nine *P. shigelloides* reference strains were sequenced using an Illumina/Solexa Genome Analyzer IIx (Illumina, USA), assembled using VelvetOptimiser v2.2, and identified by BLAST search against the NCBI Prokaryotic Genome Annotation Pipeline. For genes involved in sugar synthesis, the UniProt/SwissProt database was used. For genes involved in O-antigen processing and translocation, the program TMHMM Server v2.0 was used to confirm the characteristics of *wzx*/*wzy* or *wzm/wzt*. For genes involved in glycosyl transferase (GT), the Pfam protein motif database (http://pfam.xfam.org/) was used.

### Target Gene Selection and Phylogenetic Analysis

All the *wzx*/*wzy* sequences in this study were extracted from the O-antigen gene clusters, aligned using the ClustalW program [15], and the identity of the *wzx*/*wzy* gene was acquired using BioEdit software [16]. The phylogenetic trees based on the *wzx*/*wzy* sequences were constructed using MEGA 6.0 [17].

### Primers for the PCR Assay

The primers were designed using Primer Premier 5.0 software (Premier Biosoft International, USA) based on the sequences of the processing genes (Table 2). The formation of ring hairpin structures, self-dimers, and cross-dimers was avoided. The specificity of primers was evaluated by BLAST alignments against the GenBank database [18]. The length of the primers was about 25 nt, and Tm was between 55 and 60°C. The length of the generated PCR product was 50–200 bp. The primers were synthesized by Invitrogen (China). The numbers and sequence information of each primer are listed in Table 2.

### Development of a Serotyping PCR Assay

The amplification reactions were carried out in a total volume of 25 μl, including 12.5 μl of 2× Premix Ex Taq (Takara, China), 1 μl of 10 μΜ forward primer, 1 μl of 10 μΜ reverse primer, 1 μl of DNA, and 10.5 μl of ddH2O. The reaction parameters were as follows: 95°C for 3 min; 35 cycles of 95°C for 30 s, 60°C for 30 s, and 72°C for 1 min; followed by a final extension at 72°C for 5 min. A 2 μl aliquot of the PCR product was visualized by 1.5% agarose gel electrophoresis, and the images were captured digitally using the Gel Image System (Tanon 3500, China).

### Sensitivity with Genomic DNA

To analyze the sensitivity of the PCR assay for genomic DNA, serial ten-fold gradient dilutions of the genomic DNA of *P. shigelloides* O61 genomic DNA (100 ng, 10 ng, 1 ng, 0.1 ng, 0.01 ng, 1 pg, 0.1 pg, and 0.01 pg) were used as templates for PCR amplification to determine the minimum detection level.

### GenBank Accession Numbers

The nine O-antigen gene clusters of *P. shigelloides* serogroups O8, O17, O18, O37, O38, O39, O44, O45, and O61 have been submitted to GenBank with the accession numbers MN115821–MN115829, respectively.

## Results

### Identification of O-Antigen Gene Clusters in Nine *P. shigelloides* Genome Sequences

**Fig. 1:**
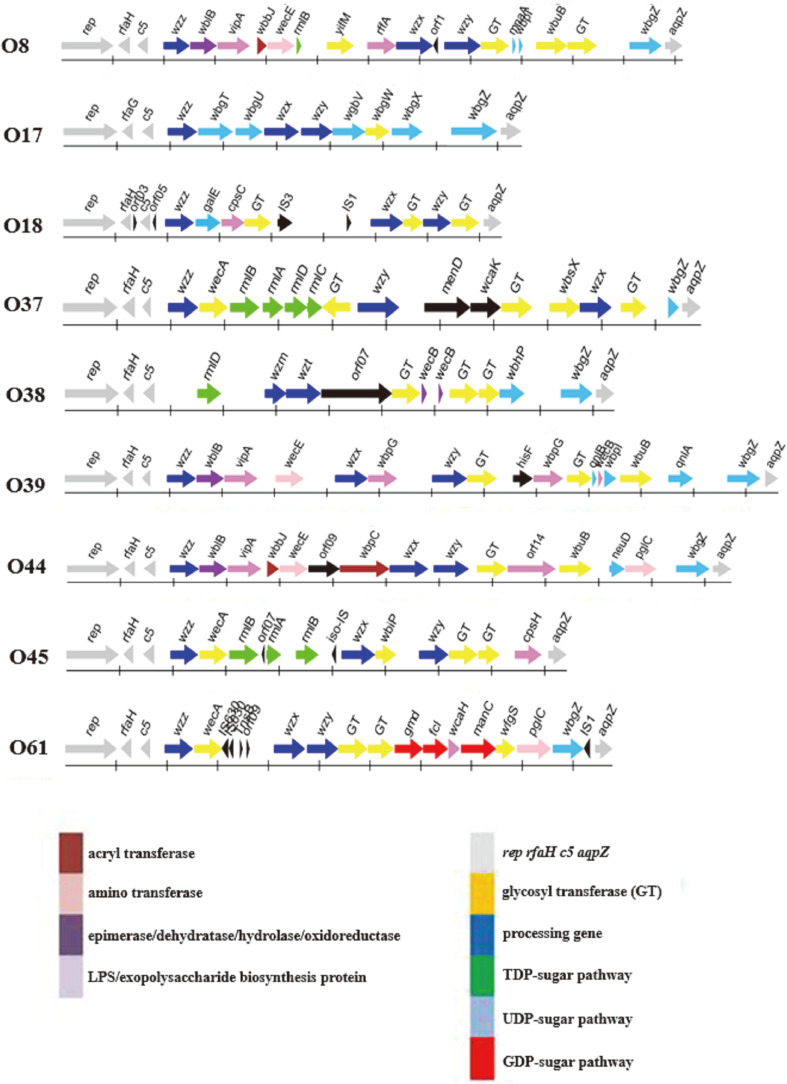
The O-antigen gene clusters of nine *P. shigelloides* serogroups O8, O17, O18, O37, O38, O39, O44, O45 and O61.

**Fig. 2:**
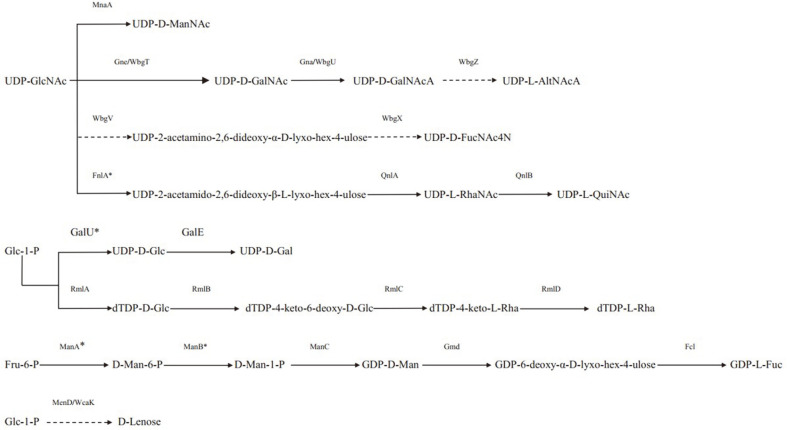
Proposed biosynthesis pathways for sugars in the nine *P. shigelloides* serogroups clusters. *Indicates the genes that were found outside the OPS clusters. Putative pathways are denoted by a broken line. MnaA, UDP-Nacetylglucosamine- 2-epimerase; Gne, UDP-N-acetylglucosamine-4-epimerase; Gna, UDP-GalNAcA synthetase; WbgZ, C-5 epimerase; FnlA*, 4,6-dehydratase, 3- and 5-epimerase; QnlA, dTDP-4-dehydrorhamnose reductase; QnlB, C-2 epimerase; GalU*, UTP-glucose-1-phosphate uridylyltransferase; GalE, UDP-glucose-4-epimerase; RmlA, glucose-1-phosphate thymidylyltransferase; RmlB, dTDP-D-glucose 4,6-dehydratase; RmlC, dTDP-4-keto-6-deoxy-Dglucose3,5-epimerase; RmlD, dTDP-6-deoxy-L-mannose-dehydrogenase. ManA*, phosphomannose isomerase; ManB*, phosphomannomutase; ManC*, mannose-1-phosphate guanylyltransferase; Gmd, GDP-mannose 4,6-dehydratase; Fcl, GDP-L-fucose synthetase. DManNAc, 2-acetamido-2-deoxy-D-mannose; D-GalNAc, 2-acetamido-2-deoxy-D-galactose; D-GalNAcA, 2-Acetamido-2- deoxy-D-galacturonic acid; L-AltNAcA,2-Acetamido-2-deoxy-L-altruronic acid; D- FucNAc4N, 2-Acetamido-4-amino-2, 4- dideoxy-D-fucose; L-RhaNAc, 2-acetamido-2,6-dideoxy-L-mannose (N-acetyl-L-rhamnosamine); D-GlcNAc, 2-acetamido- 2-deoxy-D-glucose; L-QuiNAc, 2-Acetamido-2-deoxy-L-quinovose (2-acetamido-2,6-dideoxy-L-glucose); D-Glc, Dglucose; D-Gla, D-galactose; L-Rha, L-rhamnose (6-deoxy-L-mannose); L-Fuc, L-fucose; D-Man, D-mannose.

The O-antigen gene clusters of nine *P. shigelloides* serogroups were sequenced and localized in the type strains O8, O17, O18, O37, O38, O39, O44, O45, and O61 (Table 1). All nine clusters were located between *rep* and *aqpZ* in the chromosome and comprised 24,307 bp, 16,842 bp, 16,858 bp, 23,694 bp, 20,811 bp, 27,237 bp, 25,003 bp, 18,828 bp, and 21,560 bp, respectively. Twenty-one, 14, 16, 19, 15, 21, 19, 17 and 22 open reading frames (ORFs), respectively, were identified in the nine serotypes (Tables S 1-9). The functions of the genes were assigned according to their similarities with the genes with known functions in the sugar pathway and others from available databases (Figs. 1 and 2).

### O-Antigen Cluster of *P. shigelloides* O8

In the *P. shigelloides* O8 cluster, orf16 shares 81% identity with MnaA from *Vibrio cholerae*, indicating there is a putative rare sugar, ManNAc, in the structure. Orf12 shares 53% identity with Wzx from *Aeromonas hydrophila*, and orf14 shares 34% identity with Wzy in *Aminobacterium colombiense*. The presence of *wzx* and *wzy* genes suggested the Wzx/Wzy pathway for O-antigen processing in O8. O8 has four GTs, encoded by orf10, orf15, orf18, and orf19, which share 50% identity with YifM from *Marinobacter antarcticus*, 83% identity with a GT from *Vibrio ordalii*, 58% identity with WbuB from *Photorhabdus luminescens*, and 97% identity with a GT from *S. sonnei*, respectively.

### O-Antigen Cluster of *P. shigelloides* O17

For *P. shigelloides* O17, orf5 and orf6 share 100% identity with WbgT and WbgU from *P. shigelloides* and were renamed as Gne and Gna by Liu *et al*. [19]. Working together with WbgZ, a UDP-GlcNAc can be changed to a putative rare sugar, UDP-L-AltNAcA, in this serotype. Orf9 and orf11 share 99% identity with WbgV and WbgX from *S. sonnei*, indicating a putative rare sugar, UDP-D-FucNAcA, might be present in the structure. Orf7 shares 100% identity with Wzy from *P. shigelloides* and orf8 shares 99% identity with Wzx from *S. sonnei*. The set of *wzx* and *wzy* genes indicated the presence of Wzx/Wzy pathway-related O-antigen processing in O17. There is one GT encoded by the orf10, which shares 99% identity with WbgW from *P. shigelloides*.

### O-Antigen Cluster of *P. shigelloides* O18

In the *P. shigelloides* O18 cluster, orf8 shares 63% identity with GalE from *Providencia stuartii*, indicating a putative UDP-Gal in the structure in O18. Orf12 shares 45% identity with Wzx in *Proteus vulgaris* and orf14 shares 45% identity with Wzy in *Edwardsiella ictaluri*. The presence of *wzx* and *wzy* genes indicated the presence of Wzx/Wzy pathway involvement in O-antigen processing. There are three GTs encoded by orf9, orf13, and orf15, which share 99%, 97%, and 99% identity with GTs from *P. shigelloides*.

### O-Antigen Cluster of *P. shigelloides* O37

In the *P. shigelloides* O37 cluster, orf06 and orf07 share 96% and 100% identity with RmlB and RmlA from *V. cholerae*, respectively, and orf08 and orf09 share 86% and 85% identity with RmlD and RmlC in *Vibrio anguillarum*, respectively, suggesting a putative rhamnose in the structure. Furthermore, the chemical structure of *P. shigelloides* O37 reported in 2013 showed that there is a rare sugar, α-D-Lenose ((2S)-O-(4-oxopentanoic acid)-a-D-Glc*p*), which has not been found in nature [20]. In the cluster, orf12 shares 60% identity with MenD from *Vibrio mimicus*, and orf13 shares 84% identity with WcaK from *Photobacterium* sp. These two proteins work together to catalyze D-Glc to the rare sugar D-lenose. Orf10 and orf16 share 30% identity with Wzy and Wzx from *E. coli*. The presence of the *wzx* and *wzy* genes suggests that the Wzx/Wzy pathway is involved in O-antigen processing in O37. There are four GTs encoded by orf10, orf14, orf15, and orf17, which share 65% identity with a GT from *E. coli*, 48% identity with a GT from *Shewanella loihica*, 53% identity with WbsX from *Flexilinea flocculi*, and 35% identity with a GT from *Cytophaga hutchinsonii*, respectively.

### O-Antigen Cluster of *P. shigelloides* O38

In the *P. shigelloides* O38 cluster, orf04 shares 87% identity with RmlD from *A. hydrophila*; however, whether rhamnose is present in the structure cannot be predicted using the presence of the *rmlD* gene only. Orf5 shares 62% identity with Wzm from *Aeromonas veronii*, and orf6 shares 42% identity with Wzm from *Legionella santicrucis*; therefore, the set of *wzm* and *wzt* genes suggests the presence of the transporter pathway in O38. There are three GTs encoded by the orf8, orf11, and orf12, which share 89% identity with a GT from *Aeromonas salmonicida*, 36% identity with a GT from *V. cholerae*, and 73% identity with a GT from *A. hydrophila*, respectively.

### O-Antigen Cluster of *P. shigelloides* O39

In the *P. shigelloides* O39 cluster, orf19 and orf15 share 75% identity with QnlA from *Vibrio* sp. and 98% identity with QnlB from *V. cholerae*, respectively, suggesting a putative rare sugar, UDP-L-QuiNAc, in the structure. Orf8 shares 33% identity with Wzx from *E. coli*, and orf10 shares 55% identity with Wzy from *V. cholera*. Therefore, the set of *wzx* and *wzy* genes suggests that the Wzx/Wzy pathway is involved in O-antigen processing. There are three GTs encoded by orf11, orf14, and orf18, which share 61% identity with a GT from *Pseudomonas saudiphocaensis*, 82% identity with a GT from *A. veronii*, and 89% identity with WbuB from *V. cholerae*.

### O-Antigen Cluster of *P. shigelloides* O44

In the *P. shigelloides* O44 cluster, no rare sugar could be predicted. Orf11 and orf12 share 35% and 80% identity with Wzx from *Moorella sp*. and Wzy from *V. cholerae*, respectively; therefore, the set of *wzx* and *wzy* genes suggests the presence of the Wzx/Wzy pathway in O-antigen processing. There are two GTs: Orf13 shares 55%identity with a GT from *Pseudomonas mosselii*, and orf15 shares 50% identity with a GT from *V. cholerae*.

### O-Antigen Cluster of *P. shigelloides* O45

In the *P. shigelloides* O45 cluster, orf6, orf8, and orf9 share 89% identity with RlmB from *P. shigelloides*, 92%identity with RlmA from *Aeromonas piscicola*, and 66% identity with RlmB from *A. hydrophila*, respectively, which indicates a putative rhamnose in the structure. Orf11 and orf13 share 38% and 38% identity with Wzx from *Pseudomonas* sp. 286 and Wzy from *E. coli*, respectively; thus, the set of *wzx*, *wzy*, and *wzz* genes suggests the presence of the Wzx/Wzy pathway in O-antigen processing. There are three GTs encoded by orf12, orf14, and orf15, which share 44% identity with WbiP from *Salmonella enterica*, 44% identity with a GT from *Vibrio ishigakensis*, and 48% identity with a GT from *Citrobacter freundii*, respectively.

### O-Antigen Cluster of *P. shigelloides* O61

In the *P. shigelloides* O61 cluster, orf14, orf15, and orf17 share 94% identity with Gmd from *Aeromonas caviae*, 87% identity with Fcl from *A. hydrophila*, and 75% identity with ManC from *Providencia alcalifaciens*, respectively, which indicates a putative rhamnose in the structure. Orf10 shares 35% identity with Wzx from *E. coli*, and orf11 shares 32% identity with Wzy from *P. vulgaris*; thus, the set of *wzx* and *wzy* genes indicates the presence of the Wzx/Wzy pathway in O-antigen processing. There are three GTs: orf12 and orf13 share 58% and 64% identity with GTs from *E. coli*, and orf18 shares 61% identity with WfgS from *Vibrio vulnificus*.

### Comparative Analysis of the O-Antigen Gene Clusters of O39 and O44

**Fig. 3:**
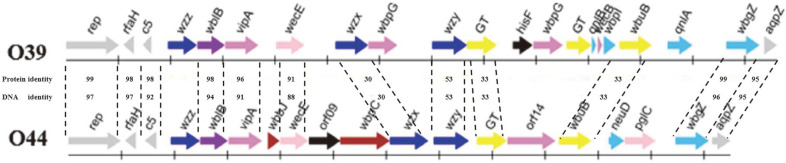
Comparison of the O-antigen gene clusters of *P. shigelloides* O39 and O44.

The comparison of the O-antigen gene clusters of O39 and O44 showed that except for the conservative and anchor genes of *rep, rfaH, c5* and *aqpZ*, there are some other genes shared by both clusters (Fig. 3). For example, both occupy *wzx*, *wzy* and *wzz*, and Wzx from O39 shares 30% identity with that from O44; Wzy, 53%; and Wzz, 42%. Also, the three GTs encoded by orf11, orf14, and orf18 in O39 cluster were blasted with the two GTs encoded by orf13 and orf15 in O44. We found that the GT of orf11 of O39 shares 33% identity with that of orf13 of O44, while the GT of orf18 of O39 shares 33% identity with that of orf15 of O44, which goes by the name of WbuB. By the number of GTs, we speculate that there are at least four sugars in the structure of O39, including the putative rare sugar UDP-L-QuiNAc represented by QnlA and QnlB; while O44 has three sugars. Moreover, WblB of O39 shares 98% identity with that of O44; VipA, 96%; WecE, 91%; and WbgZ, 99%.

### Phylogenetic Analysis

**Fig. 4:**
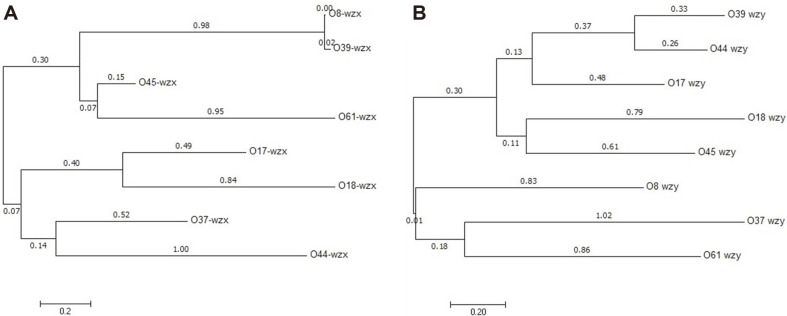
Phylogenetic analysis. An unrooted phylogenetic tree constructed by the neighbor-joining method based on the *wzx* (**A**) and *wzy* (**B**) genes is shown. Bootstrap values were based on 1000 replications and only values greater than 50% are shown.

Dendrogram analysis of *wzx* was used for eight *P. shigelloides* serogroups (O8, O17, O18, O37, O39, O44, O45, and O61 (O38 does not have *wzx*) (Fig. 4). The phylogenetic tree showed two major clades, the upper one includes four serogroups of O8, O39, O45, and O61, and the lower one includes the other four serogroups of O17, O18, O37, and O44. In the upper clade, the distance of O8 is the same as that of O39, and both are close to the two-member group of O45 and O61. In the lower clade, there are two parallel groups; O17 is grouped together with O18, and O37 is grouped together with O44 (Fig. 4A).

The *wzy* gene-based phylogenetic tree showed two major clades, the bigger one includes the five serogroups of O39, O44, O17, O18, and O45, and the smaller one includes the three serogroups of O8, O37, and O61. In the bigger clade, there are two subclades. The upper subclade consists of O17, O39, and O44, in which O39 is close to O44 and is grouped together with O17; the lower subclade consists of O18 and O45. In the smaller clade, O37 is close to O61 and is grouped together with O8 (Fig. 4B).

### Target Genes and PCR Serotyping with Specific Primers

In our previous studies we suggested that the genes involved in O-antigen synthesis, either *wzx* and *wzy*, or *wzm* and *wzt*, are highly specific to individual O serotypes [8]. Therefore, they were selected as the target genes for the development of a molecular serotyping method. Among the nine O-antigens, O38 possesses *wzm* and *wzt*, and the other eight possess *wzx* and *wzy*. Accordingly, specific primers were designed based on the *wzx* genes of O17, O18, O37, O44, and O45; the *wzy* genes of O8 and O39; the *wzt* gene of O38; and the fcl gene of O61.

**Fig. 5:**
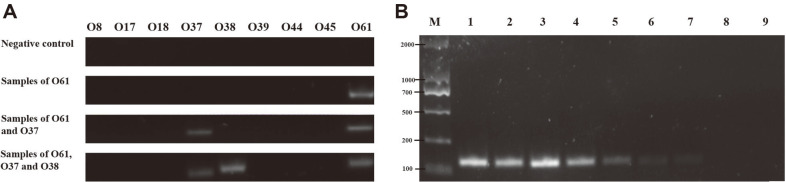
PCR detection and sensitivity. **A**. Line 1: Layout of the nine single-plex PCR with specific primers for each serotype. Line 2: Negative control. Line 3: Specificity of the PCR for *P. shigelloides* O61 without cross reaction with the other eight serogroups. Line 4: Mock sample with two serotypes of *P. shigelloides* 37 and O61. Line 5: Mock sample with three serotypes of *P. shigelloides* O37, O38 and O61. **B**. Sensitivity of detection with the genomic DNA of *P. shigelloides* O61. M, DL 500 bp Marker; Lane 1: 100 ng; Lane 2: 10 ng; Lane 3: 1 ng; Lane 4: 0.1 ng; Lane 5: 0.01 ng; Lane 6: 1 pg; Lane 7: 0.1 pg; Lane 8: 0.01 pg; and Lane 9: negative control.

After optimization, a nine singleplex PCR assay with 9 primer pairs specific to the nine *P. shigelloides* serogroups was established (Table 2). The interpretation is based on the presence or absence of the characteristic PCR amplicons (~100 - 150 bp in length) for the serotype; for example, O61 can only generate PCR product with its own specific primer pair with 100% specificity and accuracy (Figs. 5A and S1). Samples isolated from the environment usually comprise more than one serotype. The mock samples containing two (O37 and O61) and three serotypes (O37, O38 and O61) were also tested with 100% specificity and accuracy (Fig. 5A). The PCR approach is useful for the simultaneous detection of multiple serotypes.

### DNA Sensitivity for PCR Assay

For genomic DNA of *P. shigelloides* O61, serial ten-fold dilutions ranging from 100 ng to 0.01 pg were used to test the sensitivity of the PCR assay. The minimum detection level was determined as 0.1 pg with the genomic DNA of O61 shown in Fig. 5B.

### Detection of Clinical Isolates

Out of the total 16 clinical isolates collected, 13 were from the Chinese Center for Disease Control and Prevention Beijing, China, and the other 3 from Guangzhou Center for Disease Control and Prevention, Guangdong, China. The established serotyping PCR assay was applied to all the 16 samples and the data showed that three of them, clinical samples ps-1, ps-9, and ps-16, generated positive results as serotype O37 (Fig. S2). DNA sequencing of the PCR fragments confirmed the results (Fig. S3).

## Discussion

Of the nine novel O-antigens, only the O37 chemical structure, which possesses the rare sugar D-Lenose that has not been found in the nature, has been reported [20]. In addition, several other rare sugars can be deduced based on the highly homologous sugar synthetic genes; for example, dTDP-L-Rha in O37 and O45; UDP-ManNAc in O8; UDP-L-AltNAcA and UDP-D-FucNAcA in O17; UDP-Gal in O18, and UDP-L-QuiNAc in O39. No rare sugars were predicted in O38 and O44, however.

In the O-antigen clusters of the nine *P. shigelloides* serogroups, transporter pathways encoded by *wzm/wzt* are employed for O-antigen processing by O38 and the *wzt* gene was selected as its target; Wzx/Wzy pathways encoded by *wzx*/*wzy* are employed for the other eight serogroups (O8, O17, O18, O37, O39, O44, O45, and O61). Among them, *wzx* genes were selected as the targets for five serogroups (O17, O18, O37, O44, and O45). In the *wzx* dendrogram tree, O8 and O39 are almost identical; however, in the *wzy* dendrogram tree, they are far from each other and therefore the *wzy* genes were selected as targets for both of them.

Interestingly, the eight O-antigens with Wzx/Wzy pathways possess not only *wzx* and *wzy*, but also wzz, which is the gene downstream of *c5* in the cluster. Wzz is a well-known regulator of O-antigen chain-length [21]. Generally *wzz* is located within the chromosome but outside of the O-antigen clusters, as in the case of *Shigella* [19], *Salmonella* [9], *Citrobacter* [22], *Providencia* [23], and *A. hydrophila* [24]. However, together with the previous publication of 12 *P. shigelloides*, 17 of the 21 O-antigens employ the Wzx/Wzy pathway, and all 17 clusters have *wzz* genes. Similar to *P. shigelloides*, in *P. aeruginosa*, *wzz* is the next gene downstream of *himD* in the O-antigen clusters of all 20 identified serotypes [25].

In conclusion, O-antigen variability has been used as the basis for serotyping schemes established for many gram-negative bacteria, and serotyping plays a key role in detection and epidemiological surveillance. The frequent intra-species recombination events of *P. shigelloides* might lead to the expansion and diversity of serotypes which are important for understanding the evolution of this pathogen and other bacterial strains. In the present study, we described a traditional PCR procedure to serotype nine *P. shigelloides* serogroups based on their *wzx*/*wzy*/*wzt/fcl* gene sequences. Our method provides a rapid, reliable and reproducible tool for the detection, serotyping and epidemiological surveillance of *P. shigelloides*.

## Supplemental Materials



Supplementary data for this paper are available on-line only at http://jmb.or.kr.

## Figures and Tables

**Table 1:** *Plesiomonas shigelloides* strains used in this study.

*Plesiomonas shigelloides*	Lab number	Original number	Original source	Reference/Clinical Sample
O8H22	G5267	E7	Italy^a^	Salerno *et al*., 2010
O17H11	G5878	CNCTC Aer 42/89	CNCTC^b^	Maciejewska *et al*., 2013
O18H2	G6000	CNCTC Aer7007 54/89	CNCTC^b^	
O37	G5883	CNCTC Aer 39/89	CNCTC^b^	Kaszowska *et al*., 2013
O38H4	G6003	CNCTC Aer7009 59/89	CNCTC^b^	
O39H11	G6004	CNCTC Aer6410 41/89	CNCTC^b^	Maciejewska *et al*., 2013
O44H8	G6005	CNCTC Aer6416 52/89	CNCTC^b^	
O45H2	G5884	CNCTC Aer 44/89	CNCTC^b^	Maciejewska *et al*., 2013
O61H2	G5998	CNCTC Aer6999 31/89	CNCTC^b^	
Clinical isolates				
1	G6370	ps-1	CDC^c^	diarrhea
2	G6371	ps-4	CDC^c^	diarrhea
3	G6372	ps-5	CDC^c^	watery diarrhea
4	G6373	ps-6	CDC^c^	diarrhea
5	G6374	ps-7	CDC^c^	diarrhea
6	G6375	ps-8	CDC^c^	diarrhea
7	G6376	ps-9	CDC^c^	diarrhea
8	G6377	ps-16	CDC^c^	mucinous feces
9	G6378	ps-17	CDC^c^	watery diarrhea
10	G6379	ps-18	CDC^c^	diarrhea
11	G6380	ps-19	CDC^c^	diarrhea
12	G6381	ps-22	CDC^c^	diarrhea
13	G6382	ps-23	CDC^c^	watery diarrhea
14	G6383	V14-017	CDC^d^	diarrhea
15	G6384	V14-009	CDC^d^	diarrhea
16	G6385	V14-017	CDC^d^	diarrhea

^a^Department of Environmental Sciences, Parthenope University of Naples, Naples, Italy.

^b^Czech National Collection of Type Cultures (CNCTC), Czech Republic.

^c^Chinese Center for Disease Control and Prevention, Beijing, China.

^d^Guangzhou Center for Disease Control and Prevention, Guangdong, China.

**Table 2:** Primers used in this study.

Serogroup	Target gene	Primer name	Sequence（5’-3’）	Length (nt)	Tm (℃)
O8	*wzy*	wln-15653	CCACTTTATGGTTTTATCTCATCTGATT	28	58
		wln-15654	CCACTCTTGCTTTGCAACAACT	22	58
O17	*wzx*	wln-15209	AAATGACCACACCAATACAATACGA	25	58
		wln-15210	AATGAACTTTATCTTGTGTGTAGTGGAAA	29	59
O18	*wzx*	wln-15211	GGCACCTAACACTCGAGTCAAAT	23	58
		wln-15212	GGTGATGCAAGGCGTTAACTATT	23	58
O37	*wzx*	wln-15213	ATGGCTAGTGACACCTCCTGAA	22	58
		wln-15214	ACCTGGACAACACCCAACTTTA	22	58
O38	*wzt*	wln-13770	CAATTTCTTTTGCCGCTCCTA	21	58
		wln-13771	TTTTGTCAGAGAGCTGTTTTGTTGA	25	59
O39	*wzy*	wln-15659	GATGCTGGACTTTTTCTGCATAGTT	25	58
		wln-15660	AATACCAGTGTACACCACAAACCAA	25	59
O44	*wzx*	wln-15663	TTTACAGTAGTAGCAAGTCTGCGATATG	28	58
		wln-15664	TGGCCGCTTCAGTATTGCTT	20	59
O45	*wzx*	wln-15217	GATCTTGAAGTTCTCCACTATGGCTATA	28	58
		wln-15218	GCACTCTCAACTCCATCATATGAAA	25	58
O61	*fcl*	wln-15720	CCAATCTGTATGGCGAAAACG	21	59
		wln-15721	CAGGGCCGGAATCACATG	18	59
