# Supplementary material for: Genetic Analysis and Serological Detection of Novel O-Antigen Gene Clusters of Plesiomonas shigelloides
Source: J Microbiol Biotechnol. 2021 Feb 8;31(4):520–8. doi: 10.4014/jmb.2010.10008 (PMC9723277; doi:10.4014/jmb.2010.10008)
Supplement: Supplementary file 1 [file jmb-31-4-520-supple.pdf]

https://doi.org/10.4014/jmb.2010.10008

Table S1 Characteristics of the ORFs of *Plesiomonas shigelloides* O8 antigen gene cluster

ORF#	Gene name /location /direction	No. of amino acids	G+C content(%)	Similar protein/strain/GenBank accession No.	% Identity/% Similarity (No. of aa overlap)
O8-01	rep<br>(1-2031)<br>+	676	54.9	DNA helicase Rep<br>[Plesiomonas shigelloides]<br>WP_084977044	99/99(674)
O8-02	rfaH<br>(2192-2668)<br>-	158	51.8	transcription/translation regulatory transformer protein RfaH<br>[Plesiomonas shigelloides]<br>WP_039046405	98/99(157)
O8-03	c5<br>(2942-3367)<br>-	141	56.2	cytochrome c5<br>[Plesiomonas shigelloides 302-73]<br>EON89259	98/98(139)
O8-04	wzz<br>(3989-5008)<br>+	339	36.9	Wzz<br>[Plesiomonas shigelloides]<br>AAG17407	37/54 (188)
O8-05	wblB<br>(5031-6071)<br>+	346	43.7	WblB protein<br>[Vibrio albensis VL426]<br>EEO03407	84/93(322)
O8-06	vipA<br>(6093-7367)<br>+	424	40.2	Vi polysaccharide biosynthesis UDP-N-acetylglucosamine C-6 dehydrogenase TviB<br>[Aeromonas veronii]<br>WP_128814987	84/90(383)
O8-07	wbbJ<br>(7658-8023)	121	47.2	N-acetyltransferase<br>[Aeromonas cavernicola]	90/94(114)

	+			WP_100292865	
O8-08	wecE<br>(8047-9123)<br>+	358	43.4	DegT/DnrJ/EryC1/StrS family aminotransferase<br>[Vibrio cholerae]<br>WP_119566904	91/95(341)
O8-09	rmlB<br>(9207-9362)<br>+	51	60.4	dTDP-glucose 4,6-dehydratase<br>[Pseudomonas sp. GL14]<br>WP_122077650	65/82(42)
O8-10	yifM<br>(10377-11438)<br>+	353	44.2	4-alpha-L-fucosyltransferase glycosyl transferase group 56<br>[Marinobacter antarcticus]<br>SHK36675	50/68(239)
O8-11	rffA<br>(11965-13095)<br>+	376	43.2	lipopolysaccharide biosynthesis protein RffA<br>[Hydrogenimonas sp.]<br>BBG65176	71/82(309)
O8-12	wzx<br>(13092-14531)<br>+	479	36.2	O antigen flippase<br>[Aeromonas hydrophila]<br>AXL05040	53/71(307)
O8-13	orf13<br>(14546-14716)<br>-	56	40.1	hypothetical protein B9J90_06960<br>[Vibrio sp. V09_P4A23P171]<br>OXX36928	60/70(40)
O8-14	wzy<br>(14974-16404)<br>+	476	29.6	oligosaccharide repeat unit polymerase<br>[Aminobacterium colombiense]<br>WP_013049105	34/55(255)
O8-15	GT<br>(16397-17500)<br>+	367	45.3	glycosyltransferase<br>[Vibrio ordalii]<br>WP_010317085	83/90(332)

O8-16	mnaA<br>(17646-17768)<br>+	40	47.6	UDP-N-acetylglucosamine 2-epimerase (non-hydrolyzing)<br>[Vibrio cholerae]<br>WP_069647662	81/88 (32)
O8-17	wbpI<br>(17891-18040)<br>+	49	50.2	WbpI<br>[Pseudomonas aeruginosa PAO1]<br>AAC45863	59/75(37)
O8-18	wbuB<br>(18577-19794)<br>+	405	31.2	glycosyltransferase WbuB<br>[Photorhabdus luminescens]<br>WP_049584932	58/75(308)
O8-19	GT<br>(19795-20946)<br>+	383	37.7	putative glycosyltransferase<br>[Shigella sonnei]<br>CSP80792	97/98(358)
O8-20	wbgZ<br>(22225-23487)<br>+	420	44.3	wbgZ<br>[Shigella sonnei]<br>AGI19337	98/98(415)
O8-21	aqpZ<br>(23606-24307)<br>+	233	54.6	aquaporin Z<br>[Plesiomonas shigelloides]<br>WP_039046419	99/100/(232)

Table S2 Characteristics of the ORFs of *Plesiomonas shigelloides* O17 antigen gene cluster

ORF#	Gene name /location /direction	No. of amino acids	G+C content(%)	Similar protein/strain/GenBank accession No.	% Identity/% Similarity (No. of aa overlap)
O17-01	rep	676	54.8	DNA helicase Rep	100/100(676)
	(1-2031)			[Plesiomonas shigelloides]	
	-			WP_064977062	
O17-02	rfaH	158	52.2	transcription/translation regulatory transformer protein RfaH	100/100(158)
	(2186-2596)			[Plesiomonas shigelloides]	
	+			WP_064977063	
O17-03	c5	108	56.2	cytochrome c5	100/100(108)
	(2940-3380)			[Plesiomonas shigelloides 302-73]	
	+			EON89259	
O17-04	wzz	368	36.9	Wzz	93/95(351)
	(3910-5016)			[Plesiomonas shigelloides]	
	-			AAG17407	
O17-05	gna/wbgT	437	36.0	WbgT	100/100(437)
	(5052-6365)			[Plesiomonas shigelloides]	
	-			AAG17408	
O17-06	gne/wbgU	345	34.3	Vi polysaccharide biosynthesis UDP-N-acetylglucosaminuronic acid C-4 epimerase TviC	100/100(341)
	(6458-7483)			[Plesiomonas shigelloides]	
	-			WP_064977065	
O17-07	wzx	421	28.4	Wzx	100/100(421)
	(7548-8837)			[Plesiomonas shigelloides]	

	-			AAG17410	
O17-08	*wzy* (8921-10108) -	395	30.0	O-Antigen Polymerase family protein [*Shigella sonnei str.* Moseley] EJL19788	99/99 (356)
O17-09	*wbgV* (10099-11343) -	414	30.0	WbgV(plasmid) [*Shigella sonnei*] AAG17422	99/99(413)
O17-10	*wbgW* (11336-12238) -	213	26.6	WbgW [*Plesiomonas shigelloides*] AAG17413	99/99(212)
O17-11	*wbgX* (12327-13481) -	384	37.7	putative glycosyltransferase [*Shigella sonnei*] CSP80792	99/99(362)
O17-12	*wbgZ* (14532-16022) -	480	44.3	WbgZ [*Shigella sonnei*] AGI19337	98/99(477)
O17-13	*aqpZ* (16141-16842) -	233	54.6	aquaporin Z [*Plesiomonas shigelloides*] WP_039046419	99/100(233)

Table S3 Characteristics of the ORFs of *Plesiomonas shigelloides* O18 antigen gene cluster

ORF#	Gene name /location /direction	No. of amino acids	G+C content(%)	Similar protein/strain/GenBank accession No.	% Identity/% Similarity (No. of aa overlap)
O18-01	*rep*<br>(1-2031)<br>-	676	54.8	DNA helicase Rep<br>[*Plesiomonas shigelloides*]<br>WP_064977062	99/99(671)
O18-02	*rfaH*<br>(2184-2595)<br>+	136	51.8	transcription/translation regulatory transformer protein RfaH<br>[*Plesiomonas shigelloides*]<br>WP_039046405	97/98(134)
O18-03	*orf03*<br>(2704-2821)<br>-	38	65.8		
O18-04	*c5*<br>(2947-3337)<br>+	129	56.2	cytochrome c5<br>[*Plesiomonas shigelloides 302-73*]<br>EON89259	99/99(127)
O18-05	*orf05*<br>(3438-3558)<br>+	39	69.2		
O18-06	*wzz*<br>(3916-5020)<br>-	367	36.9	Wzz<br>[*Plesiomonas shigelloides*]<br>AAG17407	92/94(343)
O18-07	*galE*<br>(5093-6047)<br>-	317	38.4	UDP-glucose 4-epimerase<br>[*Providencia stuartii*]<br>AXL96436	63/76(243)
O18-08	*cpsC*	299	48.6	exopolysaccharide biosynthesis protein	74/85(255)

	(6078-6978)<br>-			[*Aeromonas caviae*]<br>KOG94764	
O18-09	*GT*<br>(6974-7991)<br>-	338	35.1	glycosyl transferase, partial<br>[*Plesiomonas shigelloides*]<br>PVU65474	99/99(191)
O18-10	*IS3*<br>(8234-8810)<br>-	191	56.4	IS3 family transposase<br>[*Klebsiella pneumoniae*]<br>WP_101140099	89/91(167)
O18-11	*IS1*<br>(10898-11063)<br>-	54	51.6	iso-IS1 ORF1<br>[*Plesiomonas shigelloides* 302-73]<br>EON87639	98/100(54)
O18-12	*wzx*<br>(11820-13065)<br>-	414	22.7	Wzx<br>[*Proteus vulgaris*]<br>AXY99335	45/68(270)
O18-13	*GT*<br>(13075-13846)<br>-	256	32.8	glycosyltransferase family 25 protein<br>[*Plesiomonas shigelloides*]<br>WP_116546925	97/99(254)
O18-14	*wzy*<br>(13835-14897)<br>-	353	29.4	O-antigen ligase domain-containing protein<br>[*Edwardsiella ictaluri*]<br>WP_015870668	45/63(206)
O18-15	*GT*<br>(14926-15982)<br>-	351	39.7	glycosyltransferase<br>[*Plesiomonas shigelloides*]<br>WP_116546927	99/99(350)
O18-16	*aqpZ*<br>(16156-16858)<br>-	233	54.6	aquaporin Z<br>[*Plesiomonas shigelloides*]<br>WP_039046419	99/99(232)

Table S4 Characteristics of the ORFs of *Plesiomonas shigelloides* O37 antigen gene cluster

ORF#	Gene name /location /direction	No. of amino acids	G+C content(%)	Similar protein/strain/GenBank accession No.	% Identity/% Similarity (No. of aa overlap)
O37-01	*rep*<br>(1-2031)<br>+	676	54.8	DNA helicase Rep<br>[*Plesiomonas shigelloides*]<br>WP_064977062	99/99(675)
O37-02	*rfaH*<br>(2186-2662)<br>-	158	51.8	transcription/translation regulatory transformer protein RfaH<br>[*Plesiomonas shigelloides*]<br>WP_039046405	99/100(158)
O37-03	*c5*<br>(2937-3263)<br>-	108	56.2	cytochrome c5<br>[*Plesiomonas shigelloides 302-73*]<br>EON89259	98/98(106)
O37-04	*wzz*<br>(3903-5009)<br>+	368	36.9	Wzz<br>[*Plesiomonas shigelloides*]<br>AAG17407	80/87(322)
O37-05	*wecA*<br>(5070-6116)<br>+	348	45.8	undecaprenyl-phosphate alpha-N-acetylglucosaminyl 1-phosphate transferase<br>*[Plesiomonas shigelloides]*<br>QCH03165.1	87/91(304)
O37-06	*rmlB*<br>(6217-7281)<br>+	354	47.8	dTDP-glucose 4,6-dehydratase<br>[*Vibrio cholerae*]<br>WP_057556020	96/98(350)
O37-07	*rmlA*<br>(7428-8183)	251	47.3	glucose-1-phosphate thymidylyltransferase<br>[*Vibrio cholerae*]	100/100(251)

	+			WP_000676094	
O37-08	*rmlD*	273	48.5	RmlD	86/94(257)
	(8249-9070)			[*Vibrio anguillarum*]	
	+			AAZ66344	
O37-09	*rmlC*	181	40.2	RmlC	85/91(169)
	(9070-9615)			[*Vibrio anguillarum*]	
	+			AAZ66345	
O37-10	*GT*	350	32.4	glycosyl transferase (plasmid)	65/76(269)
	(9615-10667)			[*Escherichia coli*]	
	-			APJ89374	
O37-11	*wzx*	511	33.5	polysaccharide biosynthesis protein	51/70(354)
	(10947-12482)			[*Bacteroidia bacterium 44-10*]	
	+			OJV86361	
O37-12	*menD*	569	37.9	2-succinyl-5-enolpyruvyl-6-hydroxy-3-cyclohexe ne-1-carboxylate synthase	60/78(442)
	(13422-15131)			[*Vibrio mimicus*]	
	+			WP_061051919	
O37-13	*wcaK*	372	31.0	polysaccharide pyruvyl transferase family protein	35/52(196)
	(15143-16261)			[*Escherichia coli*]	
	+			WP_001498411	
O37-14	*GT*	384	35.2	group 1 glycosyl transferase	48/66(252)
	(16276-17430)			[*Shewanella loihica*]	
	+			WP_011865211	
O37-15	*wbsX*	378	36.0	glycosyltransferase WbsX	53/69(263)
	(18069-19205)			[*Flexilinea flocculi*]	
	+			GAP41077	

O37-16	*orf16*<br>(19192-20364)<br>+	390	28.4	hypothetical protein<br>[*Salegentibacter holothuriorum*]<br>WP_079721598	36/59/(226)
O37-17	*GT*<br>(20717-21670)<br>+	317	29.5	a-glycosyltransferase<br>[*Cytophaga hutchinsonii*]<br>WP_011586247	35/58(172)
O37-18	*wbgZ*<br>(22473-22874)<br>+	133	44.3	wbgZ<br>[Shigella sonnei]<br>AGI19337	99/99(132)
O37-19	*aqpZ*<br>(22993-23694)<br>+	233	54.6	aquaporin Z<br>[*Plesiomonas shigelloides*]<br>WP_039046419	99/99(232)

Table S5 Characteristics of the ORFs of *Plesiomonas shigelloides* O38 antigen gene cluster

ORF#	Gene name /location /direction	No. of amino acids	G-C content(%)	Similar protein/strain/GenBank accession No.	% Identity/% Similarity (No. of aa overlap)
O38-01	*rep*<br>(1-2049)<br>-	682	55.4	DNA helicase Rep<br>[*Plesiomonas shigelloides*]<br>WP_039046404	99/98(675)
O38-02	*rfaH*<br>(2204-2614)<br>+	136	52.6	transcription/translation regulatory transformer protein RfaH<br>[*Plesiomonas shigelloides*]<br>WP_039046405	96/97(133)
O38-03	*c5*<br>(2953-3393)<br>+	146	57.6	cytochrome c5<br>[*Plesiomonas shigelloides 302-73*]<br>EON89259	99/99(145)
O38-04	*rmlD*<br>(5017-5904)<br>-	295	46.7	dTDP-4-dehydrorhamnose reductase<br>[*Aeromonas hydrophila*]<br>WP_029304027	87/91(270)
O38-05	*wzm*<br>(7580-8389)<br>-	269	32.0	ABC transporter permease<br>[*Aeromonas veronii*]<br>WP_100654180	62/81(205)
O38-06	*wzt*<br>(8379-9686)<br>-	435	35.6	ABC transporter ATP-binding protein<br>[*Legionella santicrucis*]<br>WP_058513537	44/62(260)
O38-07	*orf07*<br>(9700-12396)<br>-	58	32.4	methyltransferase<br>[*Rhizobiales bacterium*]<br>WP_112732895	81/90(812)
O38-08	*GT*	356	21.8	glycosyltransferase	80/89(319)

	(12389-13459)<br>-			[*Aeromonas salmonicida*]<br>WP_125729283	
O38-09	*wecB*<br>(13518-13712)<br>-	64	38.5	UDP-N-acetylglucosamine 2-epimerase (non-hydrolyzing)<br>[*Escherichia coli*]<br>WP_113410508	79/86(37)
O38-10	*wecB*<br>(14169-14312)<br>-	47	42.4	UDP-N-acetylglucosamine 2-epimerase (non-hydrolyzing)<br>[*Escherichia coli*]<br>WP_113410508	76/80(37)
O38-11	*GT*<br>(14579-15664)<br>-	361	35.5	glycosyltransferase<br>[*Vibrio cholerae*]<br>WP_114709334	36/55(204)
O38-12	*GT*<br>(15670-16464)<br>-	264	37.9	glycosyltransferase family 2 protein<br>[*Aeromonas hydrophila*]<br>WP_041217352	73/84(224)
O38-13	*wbhP*<br>(16461-17405)<br>-	314	38.7	UDP-glucose 4-epimerase<br>[*Vibrio cholerae*]<br>CSC16094	52/67(207)
O38-14	*wbgZ*<br>(18780-19991)<br>-	403	47.2	wbgZ<br>[*Shigella sonnei*]<br>AGI19337	98/98(398)
O38-15	*aqpZ*<br>(20110-20811)<br>-	233	55.1	aquaporin Z<br>[*Plesiomonas shigelloides*]<br>WP_039046419	99/100(233)

Table S6 Characteristics of the ORFs of *Plesiomonas shigelloides* O39 antigen gene cluster

ORF#	Gene name /location /direction	No. of amino acids	G+C content(%)	Similar protein/strain/GenBank accession No.	% Identity/% Similarity (No. of aa overlap)
O39-01	*rep*<br>(1-2031)<br>+	676	55.0	DNA helicase Rep<br>[*Plesiomonas shigelloides*]<br>WP_039046404	99/99(673)
O39-02	*rfaH*<br>(2186-2596)<br>-	136	52.1	transcription/translation regulatory transformer protein RfaH<br>[*Plesiomonas shigelloides*]<br>WP_039046405	97/98(134)
O39-03	*c5*<br>(2937-3263)<br>-	108	56.6	cytochrome c5<br>[*Plesiomonas shigelloides 302-73*]<br>EON89259	98/98(106)
O39-04	*wzz*<br>(3904-5010)<br>+	368	36.9	Wzz<br>[*Plesiomonas shigelloides*]<br>AAG17407	85/89(319)
O39-05	*wblB*<br>(5033-6073)<br>+	346	41.0	WblB protein<br>[*Vibrio albensis VL426*]<br>EEO03407	83/92(321)
O39-06	*vipA*<br>(6095-7369)<br>+	424	39.4	Vi polysaccharide biosynthesis UDP-N-acetylglucosamine C-6 dehydrogenase TviB<br>[*Vibrio cholerae*]<br>WP_119299208	83/90(385)
O39-07	*wecE*<br>(8049-9128)	359	40.0	DegT/DnrJ/EryC1/StrS family aminotransferase<br>[*Vibrio cholerae*]	87/93(334)

	+			WP_042988074	
O39-08	*wzx*<br>(10323-11570)<br>+	415	35.2	O180 family O-antigen flippase<br>[*Escherichia coli*]<br>WP_073527627	33/49(207)
O39-09	*wbpG*<br>(11572-12696)<br>+	374	32.9	putative LPS biosynthesis protein WbpG<br>[*Achromobacter insuavis AXX-A*]<br>EGP46650	49/70(250)
O39-10	*wzy*<br>(14023-15354)<br>+	443	31.7	O-antigen polysaccharide polymerase Wzy<br>[*Vibrio cholerae*]<br>ADF80999	55/73(314)
O39-11	*GT*<br>(15354-16493)<br>+	379	33.1	glycosyltransferase<br>[*Pseudomonas saudiphocaensis*]<br>WP_125837761	51/65(248)
O39-12	*hisF*<br>(17113-17877)<br>+	254	37.4	imidazole glycerol phosphate synthase subunit HisF<br>[*Vibrio cholerae*]<br>WP_069647659	87/95(241)
O39-13	*wbpG*<br>(17890-19035)<br>+	374	38.0	putative LPS biosynthesis protein WbpG<br>[*Achromobacter insuavis AXX-A*]<br>EGP46650	49/70(250)
O39-14	*GT*<br>(19172-20131)<br>+	319	44.8	glycosyltransferase<br>[*Aeromonas veronii*]<br>WP_101531179	82/89(287)
O39-15	*qnlB*<br>(20141-20299)	52	43.4	UDP-N-acetylglucosamine 2-epimerase (non-hydrolyzing)<br>[*Vibrio cholerae*]	98/100(49)

	+			WP_069647662	
O39-16	*wecB*	56	46.2	UDP-N-acetylglucosamine 2-epimerase (non-hydrolyzing)	95/96(54)
	(20365-20535)			[*Vibrio cholerae*]	
	+			WP_069647662	
O39-17	*wbpI*	153	47.2	WbpI	60/71(103)
	(20596-21057)			[*Pseudomonas aeruginosa PAO1*]	
	+			AAC45863	
O39-18	*wbuB*	407	41.0	glycosyltransferase WbuB	89/93(382)
	(21199-22422)			[*Vibrio cholerae*]	
	+			WP_084980852	
O39-19	*qnlA*	317	40.3	SDR family oxidoreductase	75/82(317)
	(23035-23988)			[*Vibrio sp*]	
	+			ADF80967	
O39-20	*wbgZ*	420	46.9	wbgZ	98/98(415)
	(25291-26553)			[*Shigella sonnei*]	
	+			AGI19337	
O39-21	*aqpZ*	165	56.0	aquaporin Z	78/78(165)
	(26740-27237)			[*Plesiomonas shigelloides*]	
	+			WP_039046419	

Table S7 Characteristics of the ORFs of *Plesiomonas shigelloides* O44 antigen gene cluster

ORF#	Gene name /location /direction	No. of amino acids	G+C content(%)	Similar protein/strain/GenBank accession No.	% Identity/% Similarity (No. of aa overlap)
O44-01	*rep*	658	55.2	DNA helicase Rep	99/99(657)
	(1-1977)			[*Plesiomonas shigelloides*]	
	+			WP_039046404	
O44-02	*rfaH*	136	51.8	transcription/translation regulatory transformer protein RfaH	98/100(136)
	(2131-2541)			[*Plesiomonas shigelloides*]	
	-			WP_039046405	
O44-03	*c5*	146	56.2	cytochrome c5	99/99(145)
	(2876-3316)			[*Plesiomonas shigelloides 302-73*]	
	-			EON89259	
O44-04	*wzz*	362	36.9	Wzz	38/56(208)
	(3867-4955)			[*Plesiomonas shigelloides*]	
	+			AAG17407	
O44-05	*wblB*	346	43.7	WblB protein	83/93(323)
	(4978-6018)			[*Vibrio albensis VL426*]	
	+			EEO03407	
O44-06	*vipA*	424	40.2	Vi polysaccharide biosynthesis UDP-N-acetylglucosamine C-6 dehydrogenase TviB	83/90(383)
	(6039-7313)			[*Aeromonas veronii*]	
	+			WP_128814987	
O44-07	*wbbJ*	145	45.3	N-acetyltransferase	90/94(136)

	(7533-7970)<br>+			[*Aeromonas hydrophila*]<br>WP_065476729	
O44-08	*wecE*<br>(7994-9070)<br>+	358	42.5	DegT/DnrJ/EryC1/StrS family aminotransferase<br>[*Vibrio cholerae*]<br>WP_042988074	89/93(334)
O44-09	*orf09*<br>(9075-10265)<br>+	396	39.3	hypothetical protein<br>[*Pseudomonas saudiphocaensis*]<br>WP_125837754	48/62(240)
O44-10	*wbpC*<br>(10262-12139)<br>+	625	41.6	acyltransferase<br>[*Vibrio cholerae*]<br>WP_084980839	65/76(476)
O44-11	*wzx*<br>(12136-13587)<br>+	483	51.1	O-antigen flippase Wzx<br>[*Moorella sp. 60_41*]<br>KUK12942	35/57(254)
O44-12	*wzy*<br>(13792-15114)<br>+	440	34.9	O-antigen polymerase<br>[*Vibrio cholerae*]<br>ADF80999	80/89(395)
O44-13	*GT*<br>(15429-16556)<br>+	375	52.2	glycosyltransferase<br>[*Pseudomonas mosselii*]<br>WP_062361543	55/70(262)
O44-14	*orf14*<br>(16570-18399)<br>+	609	37.2	aminotransferase<br>[*Aeromonas hydrophila*]<br>AXL05024	71/82(487)
O44-15	*wbuB*<br>(18518-19738)<br>+	406	32.4	glycosyltransferase WbuB<br>[*Vibrio cholerae*]<br>WP_114774246	50/66(268)

O44-16	*neuD*<br>(20407-20994)<br>+	154	46.0	putative acetyltransferase<br>[*Vibrio cholerae*]<br>ADF80986	74/84(164)
O44-17	*pglC*<br>(21000-22175)<br>+	391	43.0	DegT/DnrJ/EryC1/StrS aminotransferase family protein<br>[*Plesiomonas shigelloides*]<br>WP_010862880	97/98(384)
O44-18	*wbgZ*<br>(22921-24183)<br>+	509	44.3	wbgZ<br>[*Shigella sonnei*]<br>AGI19337	98/99(416)
O44-19	*aqpZ*<br>(24302-25003)<br>+	233	54.6	aquaporin Z<br>[*Plesiomonas shigelloides*]<br>WP_039046419	98/99(231)

Table S8 Characteristics of the ORFs of *Plesiomonas shigelloides* O45 antigen gene cluster

ORF#	Gene name /location /direction	No. of amino acids	G+C content(%)	Similar protein/strain/GenBank accession No.	% Identity/% Similarity (No. of aa overlap)
O45-01	*rep*<br>(1-1974)<br>+	657	55.2	DNA helicase Rep<br>[*Plesiomonas shigelloides*]<br>WP_039046404	99/99(656)
O45-02	*rfaH*<br>(2129-2512)<br>-	127	51.8	transcription/translation regulatory transformer protein RfaH<br>[*Plesiomonas shigelloides*]<br>WP_039046405	95/98(125)
O45-03	*c5*<br>(2875-3300)<br>-	141	56.2	cytochrome c5<br>[*Plesiomonas shigelloides 302-73*]<br>EON89259	96/98(139)
O45-04	*wzz*<br>(3918-4946)<br>+	342	36.9	Wzz<br>[*Plesiomonas shigelloides*]<br>AAG17407	38/55(192)
O45-05	*wecA*<br>(5006-6052)<br>+	348	40.7	UDP-N-acetylglucosamine--undecaprenyl-phosphate N-acetylglucosaminephosphotransferase<br>[*Plesiomonas shigelloides*]<br>WP_084977041	90/94(328)
O45-06	*rmlB*<br>(6132-7217)<br>+	361	47.5	dTDP-glucose 4,6-dehydratase<br>[*Plesiomonas shigelloides*]<br>WP_084977040	89/92(335)
O45-07	*orf07*<br>(7343-7456)<br>-	37	64.2	hypothetical protein GY12_19310<br>[*Micrococcus luteus*]<br>KFC50841	52/74(23)

O45-08	*rmlA*<br>(7529-8080)<br>+	183	45.3	RmlA<br>[*Aeromonas piscicola*]<br>ABX39497	92/95(175)
O45-09	*rmlB*<br>(8646-9479)<br>+	277	47.8	dTDP-glucose-4,6-dehydratase<br>[*Aeromonas hydrophila*]<br>AAM74477	66/81(222)
O45-10	*iso-IS*<br>(9980-10144)<br>-	54	48.5	iso-IS1 ORF1<br>[*Plesiomonas shigelloides 302-73*]<br>EON88667	83/87(47)
O45-11	*wzx*<br>(10355-11611)<br>+	418	49.0	O-antigen translocase<br>[*Pseudomonas sp. 286*]<br>WP_122852601	38/58(242)
O45-12	*wbiP*<br>(11631-12410)<br>+	259	32.4	putative glycosyl transferase<br>[*Salmonella enterica subsp. salamae*]<br>VEA60470	44/64(155)
O45-13	*wzy*<br>(13271-14377)<br>+	368	34.7	Wzy<br>[*Escherichia coli*]	38
O45-14	*GT*<br>(14394-15479)<br>+	361	31.6	glycosyl transferase<br>[*Vibrio ishigakensis*]<br>GAM59550	44/61(219)
O45-15	*GT*<br>(15495-16304)<br>+	269	30.7	glycosyltransferase family 2 protein, partial<br>[*Citrobacter freundii*]<br>RVS77081	48/66(171)
O45-16	*cpsH*<br>(16861-17886)	341	22.9	CpsH<br>[*Streptococcus iniae*]	38/56(185)

	+			AAY17300	
O45-17	*aqpZ* (18127-18828) +	233	54.6	aquaporin Z [*Plesiomonas shigelloides*] WP_039046419	99/100(233)

Table S9 Characteristics of the ORFs of *Plesiomonas shigelloides* O61 antigen gene cluster

ORF#	Gene name /location /direction	No. of amino acids	G+C content(%)	Similar protein/strain/GenBank accession No.	% Identity/% Similarity (No. of aa overlap)
O61-01	*rep*<br>(1-2031)<br>+	676	55.2	DNA helicase Rep<br>[*Plesiomonas shigelloides*]<br>WP_039046404	99/99(675)
O61-02	*rfaH*<br>(2186-2596)<br>-	158	51.8	transcription/translation regulatory transformer protein RfaH<br>[*Plesiomonas shigelloides*]<br>WP_039046405	96/99(157)
O61-03	*c5*<br>(2949-3338)<br>-	108	56.2	cytochrome c5<br>[*Plesiomonas shigelloides 302-73*]<br>EON89259	98/98(106)
O61-04	*wzz*<br>(3918-5024)<br>+	368	36.9	Wzz<br>[*Plesiomonas shigelloides*]<br>AAG17407	92/95(346)
O61-05	*wecA*<br>(5085-6182)<br>+	348	40.7	UDP-N-acetylglucosamine--undecaprenyl-phosphate N-acetylglucosaminephosphotransferase<br>[*Plesiomonas shigelloides*]<br>WP_084977041	88/93(324)
O61-06	*IS630*<br>(6165-6419)<br>-	84	47.5	IS630 transposase<br>[*Escherichia coli*]<br>SQU52860	95/97(82)
O61-07	*IS630*<br>(6437-6613)<br>-	58	53.7	IS630 transposase<br>[*Shigella sonnei*]<br>SVI91694	88/89(52)

O61-08	*TnsB*<br>(6871-6990)<br>+	39	45.2	Transposon Tn7 transposition protein TnsB<br>[*Klebsiella pneumoniae*]<br>WP_039550977	67/73(22)
O61-09	*orf09*<br>(7141-7257)<br>+	38	61.4		
O61-10	*wzx*<br>(8217-9443)<br>+	408	57.4	Wzx<br>[*Escherichia coli* O49]	35/-
O61-11	*wzy*<br>(9521-10747)<br>+	408	23.1	Wzy<br>[*Proteus vulgaris*]<br>AXY99635	32/53(139)
O61-12	*GT*<br>(10754-11911)<br>+	385	29.7	glycosyltransferase family 1 protein<br>[*Escherichia coli*]<br>WP_087895187	58/74(273)
O61-13	*GT*<br>(11921-12940)<br>+	339	35.3	glycosyltransferase family 1 protein<br>[*Escherichia coli*]<br>WP_087895188	64/77(261)
O61-14	*gmd*<br>(12981-14093)<br>+	370	53.0	GDP-mannose 4,6-dehydratase<br>[*Aeromonas caviae*]<br>WP_124813137	94/97(359)
O61-15	*fcl*<br>(14097-15077)<br>+	326	51.7	GDP-L-fucose synthase<br>[*Aeromonas hydrophila*]<br>WP_016351359	87/92(301)
O61-16	*wcaH*<br>(15090-15554)	154	40.2	GDP-mannose mannosyl hydrolase<br>[*Vibrio cholerae*]	84/91(141)

	+			WP_002030628	
O61-17	*manC*<br>(15588-16976)<br>+	462	39.5	ManC<br>[*Providencia alcalifaciens*]<br>AXL96543	75/88(405)
O61-18	*wfgS*<br>(16976-17722)<br>+	248	32.3	glycosyltransferase<br>[*Vibrio vulnificus*]<br>WP_085760706	61/80(200)
O61-19	*pglC*<br>(17796-19145)<br>+	449	49.1	phosphomannomutase<br>[*Vibrio vulnificus*]<br>WP_072600290	91/94(424)
O61-20	*wbgZ*<br>(19198-20412)<br>+	404	46.3	mannose-6-phosphate isomerase, class I<br>[*Vibrio cholerae*]<br>WP_057563067	83/90(366)
O61-21	*IS1*<br>(20430-20672)<br>-	80	46.0	IS1 transposase<br>[*Edwardsiella ictaluri*]<br>STP81392	68/85(34)
O61-22	*aqpZ*<br>(20859-21560)<br>+	233	54.6	aquaporin Z<br>[*Plesiomonas shigelloides*]<br>WP_039046419	99/100(233)

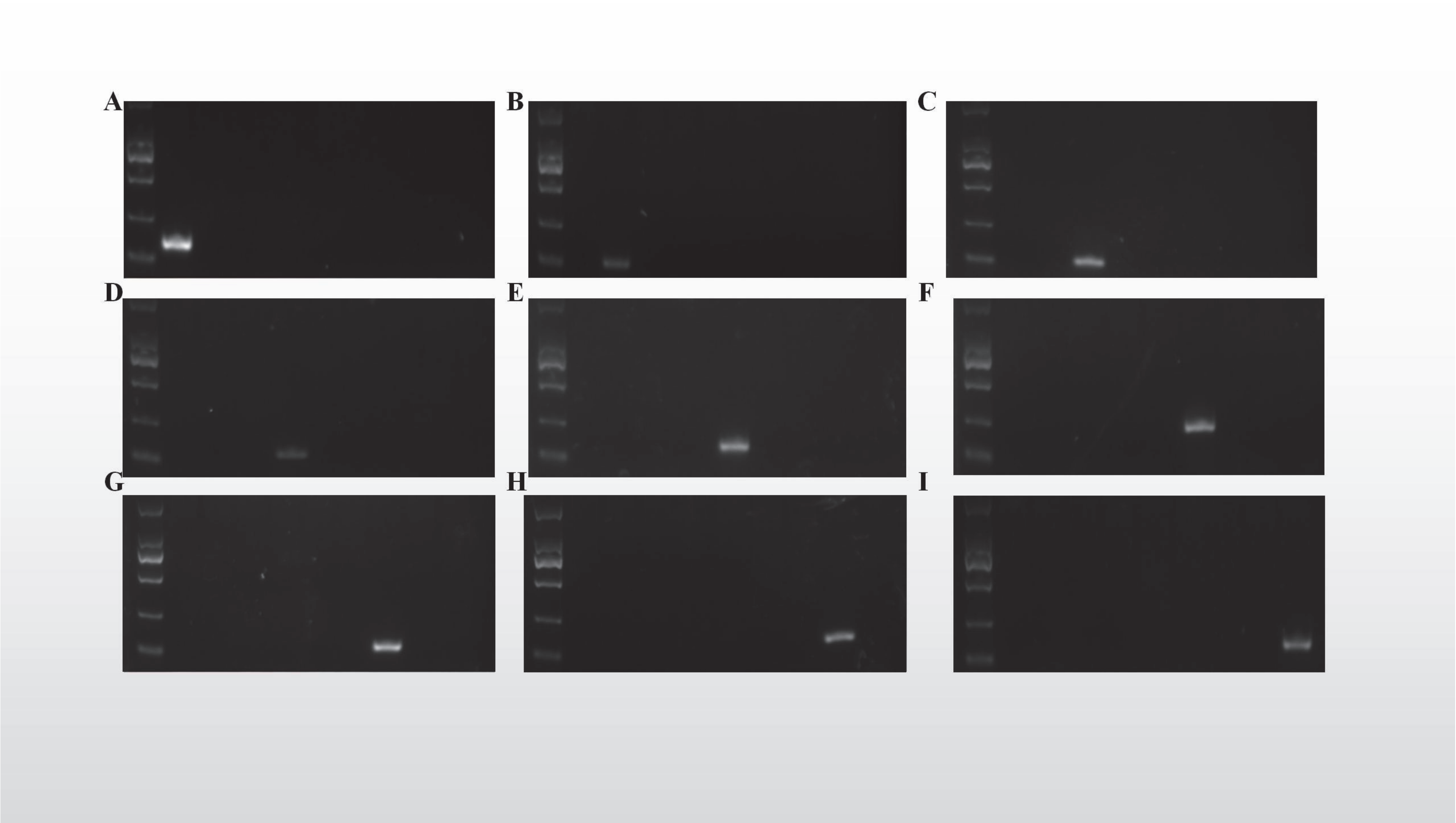

Fig. S1

1 2 3 4 5 6 7 8 9 10 11 12 13 14 15 16 17

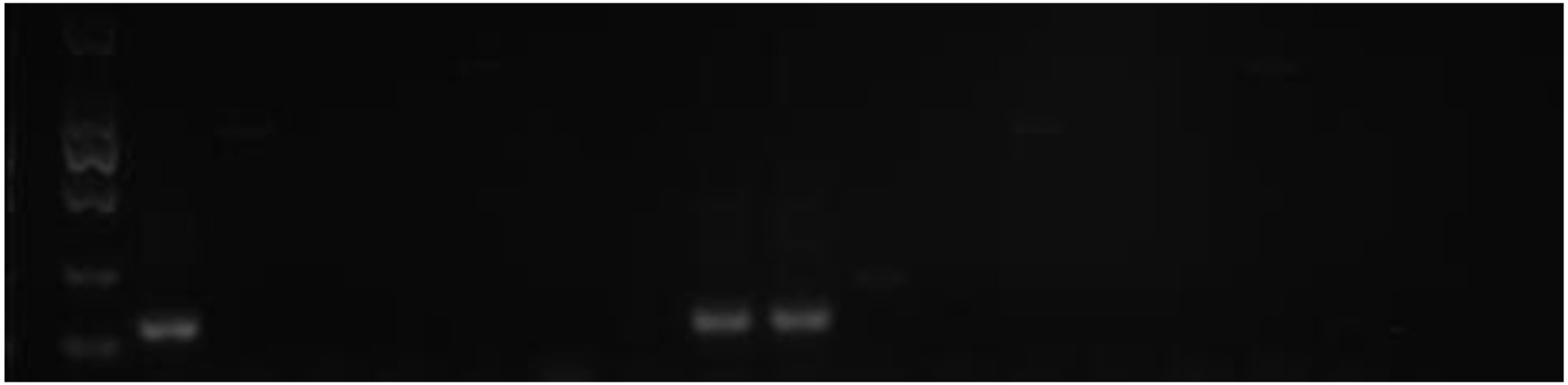

Fig. S2

A

```
ps-1  1       GACACCTCCTGAAGGTGTCATTATTTTTACTAGGCTTAGTTTGGTTGAAGTGCTTGTTAT  60
              ||||||||||||||||||||||||||||||||||||||||||||||||||||||||||||
O37   172550  GACACCTCCTGAAGGTGTCATTATTTTTACTAGGCTTAGTTTGGTTGAAGTGCTTGTTAT  172609

ps-1  61      GTCATTGAGCTTATCAATAGCAACAGCTGCGAGAGCACCAGGCAAAATGAAAGAGTATGA  120
              ||||||||||||||||||||||||||||||||||||||||||||||||||||||||||||
O37   172610  GTCATTGAGCTTATCAATAGCAACAGCTGCGAGAGCACCAGGCAAAATGAAAGAGTATGA  172669

ps-1  121     ATTAAAGTTGGGTGT  135
              |||||||||||||||
O37   172670  ATTAAAGTTGGGTGT  172684
```

B

```
ps-9  1       AACACCCAACTTTAATTCATACTCTTTCATTTTGCCTGGTGCTCTCGCAGCTGTTGCTAT  60
              ||||||||||||||||||||||||||||||||||||||||||||||||||||||||||||
O37   172685  AACACCCAACTTTAATTCATACTCTTTCATTTTGCCTGGTGCTCTCGCAGCTGTTGCTAT  172626

ps-9  61      TGATAAGCTCAATGACATAACAAGCACTTCAACCAAACTAAGCCTAGTAAAAATAATGAC  120
              ||||||||||||||||||||||||||||||||||||||||||||||||||||||||||||
O37   172625  TGATAAGCTCAATGACATAACAAGCACTTCAACCAAACTAAGCCTAGTAAAAATAATGAC  172566

ps-9  121     ACCTTCAGGAGGTGTCA  137
              |||||||||||||||||
O37   172565  ACCTTCAGGAGGTGTCA  172549
```

C

```
ps-16  1       AACACCCAACTTTAATTCATACTCTTTCATTTTGCCTGGTGCTCTCGCAGCTGTTGCTAT  60
               ||||||||||||||||||||||||||||||||||||||||||||||||||||||||||||
O37    172685  AACACCCAACTTTAATTCATACTCTTTCATTTTGCCTGGTGCTCTCGCAGCTGTTGCTAT  172626

ps-16  61      TGATAAGCTCAATGACATAACAAGCACTTCAACCAAACTAAGCCTAGTAAAAATAATGAC  120
               ||||||||||||||||||||||||||||||||||||||||||||||||||||||||||||
O37    172625  TGATAAGCTCAATGACATAACAAGCACTTCAACCAAACTAAGCCTAGTAAAAATAATGAC  172566

ps-16  121     ACCTTCAGGAGGTGTCA  137
               |||||||||||||||||
O37    172565  ACCTTCAGGAGGTGTCA  172549
```

Fig. S3
